# Pedestrians’ Perceptions of Motorized Traffic in Suburban–Rural Areas of a Metropolitan Region: Exploring Measurement Perspectives

**DOI:** 10.3390/ijerph23020206

**Published:** 2026-02-06

**Authors:** Dan Andersson, Lina Wahlgren, Peter Schantz

**Affiliations:** 1 The Research Unit for Movement, Health and Environment, Department of Physical Activity and Health, The Swedish School of Sport and Health Sciences, GIH, 114 86 Stockholm, Sweden; dan.andersson@hb.se (D.A.); lina.wahlgren@gih.se (L.W.); 2 Faculty of Caring Science, Work Life and Social Welfare, University of Borås, 501 90 Borås, Sweden

**Keywords:** walking, environment, motorized vehicles, pedestrians, suburban, rural, active commuting

## Abstract

**Background**: Since World War II, the number of motorized vehicles has increased dramatically. Yet, few studies have evaluated how perceptions of single and multiple motorized traffic variables, in different combinations, influence pedestrians’ appraisals of the route environment in relation to whether it facilitates or deters walking. We have previously illuminated this in an inner urban area of a metropolitan region. This study aims to scrutinize these matters in the suburban–rural parts of the same metropolitan area. For comparative reasons, we use the same methods as used for the inner urban area. Our hypothesis is that these kinds of perceptions, to some extent, may be context-specific. **Methods**: Relations between pedestrians’ perceptions of motorized traffic variables (flow and speeds of motor vehicles, noise, and exhaust fumes) and combinations of them, as well as if appraisals of route environments hinder–stimulate walking and are unsafe–safe for reasons of traffic, have been evaluated. This was studied in the suburban and rural areas of Greater Stockholm, Sweden. The pedestrians (n = 233) rated their route environment with the Active Commuting Route Environment Scale (ACRES). Correlation, multiple regression, and mediation analyses were used to study the relationships. **Results**: The regression analyses showed that noise was the primary negative predictor variable in relation to hindering–stimulating walking. With respect to the other outcome, unsafe–safe traffic, none of the variables had a significant relation. The mediation analyses showed that (1) vehicle speed had an indirect effect on unsafe–safe traffic via noise, (2) both vehicle speed and vehicle flow had, via noise, indirect effects on hinders–stimulates walking, and (3) vehicle speed had, via vehicle flow, an indirect effect on noise and exhaust fumes. **Conclusions**: In suburban–rural route environments, noise protrudes as a hindering variable for walking. The mediation analyses showed that vehicle speed intensified noise and had negative effects on both outcomes. Therefore, by reducing vehicle speed, noise levels will be lowered, and the walking experience is likely to be enhanced, which can influence the amount of walking. The results are further illuminated through the exploration of existing and potential future research strategies.

## 1. Introduction

Walking is a common form of locomotion, and humans walk for various purposes, such as transportation. The health benefits of walking are well recognised [1,2,3], and active transportation can contribute to sustainable mobility [4].

However, if people are to walk more, it is important that pedestrian environments support this form of movement. Based on pedestrians’ reports, the presence of trees along streets and wide sidewalks enhances well-being, whereas traffic noise, deteriorated sidewalks, and difficult crossings hinder walking, particularly for older adults and women [5]. There are also reports that pedestrians favour routes perceived as green and blue, which have less traffic and are considered safer, even when these are longer than the most direct options [6]. Furthermore, pedestrians who perceive that their routes become less pleasant over time reduce their walking [7]. Typically, depending on the research strategies used in these kinds of studies, it is difficult to precisely pinpoint the specific variable(s) that are critical for existing walking behaviours.

Since World War II, the number of motorized vehicles has increased dramatically. This has profoundly reshaped our cities [8] (p. 133). Since walking is common in metropolitan areas, it is essential to investigate how the transformed metropolitan environment, characterized by an increased number of motorized vehicles, affects pedestrians in terms of individual traffic variables, such as vehicle flow, speed, noise, and exhaust fumes.

Numerous environmental assessment tools related to walkability have been developed. Examples of such tools are the Neighbourhood Environment Walkability Scale (NEWS), the Instruments for Assessing Levels of PHysical Activity and fitness questionnaire (ALPHA), and the Perceptions of the Environment in the Neighbourhood Scale (PENS). These tools have questions about the built environment, primarily with four- or five-point response scales of the Likert type, in relation to physical activity.

The NEWS evaluates residents’ perception of neighbourhood design features associated with physical activity [9]. The ALPHA instrument examines environmental factors related to physical activity in a European context [10,11], drawing inspiration from other questionnaires, such as the NEWS. The PENS was developed to assess adults’ perceptions of their neighbourhood environment, with items selected and adapted from the ALPHA instrument [12].

The above-mentioned instruments have a focus on the residential area, often defined as the area that can be walked to within 10–15 min [11,12]. Furthermore, a limitation of ordinal scales, such as Likert scales, is that the response options are not evenly spaced, which can limit the use of parametric tests that require data measured on an interval or ratio scale.

To fully grasp the effect of motorized traffic variables on walking behaviour is challenging. A holistic approach that captures perceptions of multiple variables is likely to provide insights that can be overlooked when considering only one or two variables in isolation. Additionally, the absence of a clear and consistent terminology makes comparisons between studies difficult. For example, in one study, the following items were used: “Crossing busy roads is a big problem” and “Traffic makes it dangerous or unpleasant” [13]. How should these items be interpreted? What is the actual problem: vehicle speed, traffic flow, noise, exhaust fumes, or a combination of these? Or is there another variable at play?

Instead of focusing on the behaviour of walking, e.g., the amount of walking, a more accessible way forward could be to analyze how various traffic variables are perceived, and route environments appraised by those who walk regularly in such settings.

**Figure 1:**
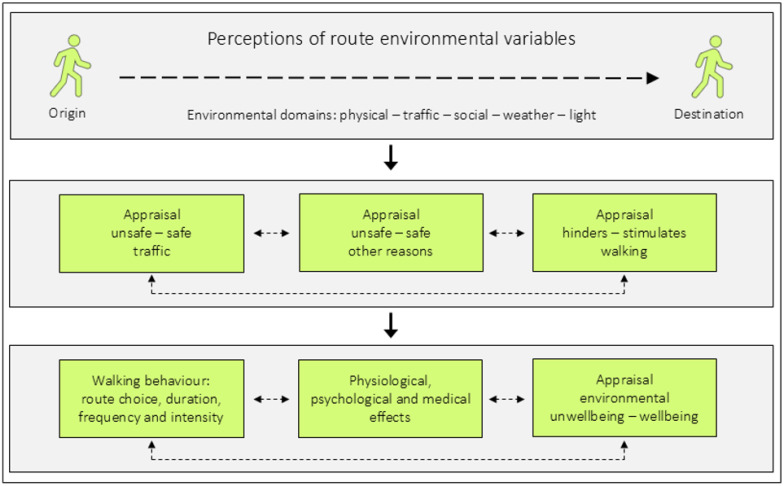
The route environments consist of several environmental domains: physical (stationary objects), traffic (mobile objects), social (individual interactions), weather (wind, rain, sun, etc.), and light conditions (natural and artificial light). These domains represent a number of predictor variables, and perceptions of them can influence safety appraisals as well as perceptions of whether the environment hinders or stimulates walking. These appraisals can impact walking behaviour, physiological, psychological, and medical effects, as well as environmentally induced unwellbeing–wellbeing. The bidirectional lines indicate potential mutual relationships. The background to this conceptual framework is described in a previous study [15] (pp. 26–29). Figure 1 is adapted from material previously published in three publications (cf. [15,16,17]).

Below is a conceptual model illustrating how perceptions of route environmental variables can influence pedestrians, modified from Schantz, [14] (p. 150), see Figure 1.

From our perspective, it is essential to distinguish the various roles and relationships between four variables related to motorized traffic: speed, flow, noise, and exhaust fumes, and to examine how they are linked to the outcomes of unsafe or safe traffic as well as hindering or stimulating walking.

**Figure 2:**
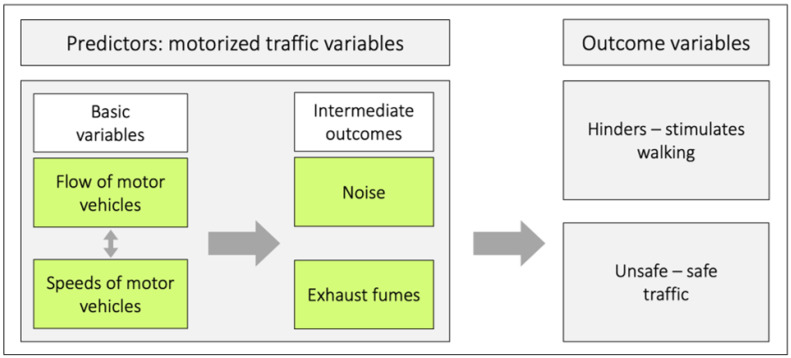
The basic variables give rise to the intermediate outcomes. All the variables can, in principle, relate independently or in various combinations to the two outcome variables. *Flow of motor vehicles* refers to the number of vehicles passing a specific reference point during a defined time period. The bidirectional arrow between the basic variables indicates that there is a relationship between them. Under conditions of uninterrupted flow, *flow*, *vehicle density* (vehicles per km), and *speed* are all interrelated: q = k v, where q represents *flow*, k represents *density*, and v represents *speed*. Figure 2 has previously appeared in three publications (cf. [15,16,17]).

Figure 2 illustrates a conceptual model that shows the relationships among the four motorized traffic variables, both internally and in relation to the outcomes of hindering or stimulating walking as well as unsafe or safe traffic. This model can be used to analyze the different roles and relations among these four motorized traffic variables in the context of walking. We consider the *speeds of motor vehicles* and the *flow of motor vehicles* as basic variables, and *noise* and *exhaust fumes* as intermediate outcomes.

We have previously studied how motorized traffic variables are perceived among those who commute by foot in the inner urban area of Stockholm, Sweden [16]. In that area, *noise* related negatively to both *hinders–stimulates walking* and *unsafe–safe traffic*, whereas *vehicle speed* is negatively related to *unsafe–safe traffic*.

The external validity of those results, however, remains unexplored in relation to pedestrians. The importance of such a perspective is underlined by the fact that in studies of cyclists in the suburban–rural areas of Greater Stockholm, Sweden, it has been noted that both *vehicle flow* and *noise* were negatively related to *inhibits–stimulates* cycling [18]. This contrasts with findings from cyclists in the inner urban area, in which only the factor *exhaust fumes* was negatively related to *inhibits–stimulates* cycling [19]. Whether such discrepancies also exist among pedestrians is, to our knowledge, unknown.

Furthermore, it is essential to understand the rationale behind studying different environments, as discussed in [20]. To state this rationale in simple terms: there are good reasons to believe that the same objective levels of motorized vehicle flows, speeds, noise, and exhaust fumes can lead to different levels of perceptions depending on the surrounding environment for the traffic.

**Figure 3:**
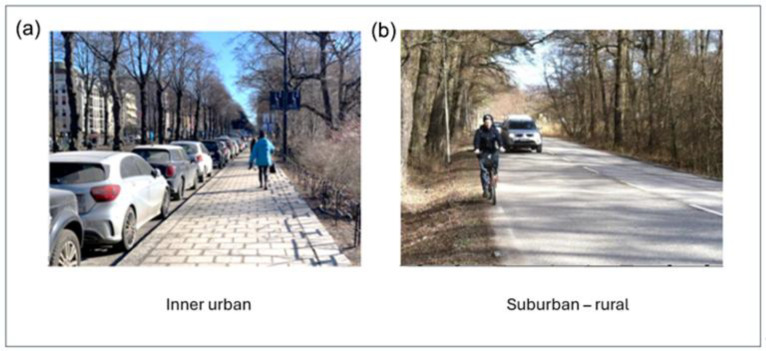
(**a**,**b**) The effect of environmental variables, such as a given flow and speed of motorized vehicles, on pedestrians can differ depending on, e.g., parked cars or distance to the motorized vehicles in inner urban vs. suburban–rural areas. Figure 3a,b have previously appeared [15].

Figure 3a,b show photos from the inner urban vs. suburban–rural study areas in Greater Stockholm. Cars are often parked along streets in inner urban areas, and will thereby sometimes hide the vehicle flow and create distance to the pedestrians. However, more or less noise will still reach those walking on the pavements. In the suburban–rural area, the encounter between pedestrians and traffic can differ significantly, and it is overall quite distinct from that in the inner urban area.

**Figure 4:**
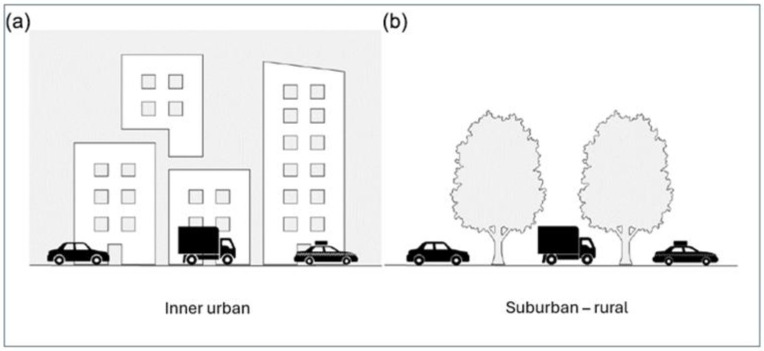
(**a**,**b**) The effect on pedestrians of given levels of all environmental variables associated with motorized vehicles can differ depending on the overall context, specifically whether it is built-up versus natural settings, and whether the area is inner urban, suburban, or rural. Figure 4a,b have previously appeared in [15].

Figure 4a,b illustrate other differences related to how traffic variables can be perceived within the context in which the traffic occurs. These differences are related to the greater and higher building volumes found near the streets in the inner urban area, compared to the more common gradient of lower houses and smaller housing volumes, or none at all, in the suburban–rural area.

Three aspects of potential differences will be stated here. The first relates to how the same traffic flow is perceived depending on its background, the second concerns the reflection of sounds from house façades, and the third concerns the accumulation of exhaust fumes when open-air spaces around traffic flows are limited by built-up settings.

The aim of the present study is therefore to investigate, in a suburban–rural setting, the relationships between the four motorized traffic variables, the basic variables, and the intermediate outcomes, as well as how these variables, and their combinations, relate to the outcome variables.

We have studied male and female walking commuters (*n* = 233) in the suburban–rural area of Greater Stockholm, Sweden, for these purposes. The pedestrians rated their commuting routes using the Active Commuting Route Environment Scale (ACRES), a self-report tool that assesses pedestrian commuters’ perceptions and appraisals of their commuting route environments [21,22].

Given the importance of gaining knowledge about these issues, we will explore current and potential future research strategies in the Section 4.

The public health relevance of this study is three-fold: (i) it is important to understand how environmental variables affect the intermediate outcomes of unsafety–safety for reasons of traffic as well as if it is hindering–stimulating the walking since these variables relate to environmental unwellbeing–wellbeing, which is a variable of direct health relevance; (ii) whether the environmental variables affect the behaviour of walking, then the level of physical activity stands for another dimension of public health; (iii) the third dimension relates to if walking is substituting for motorized transport which stand for wider negative effects on population health through, e.g., noise, exhaust fumes or CO_2_ emissions.

## 2. Method

### 2.1. Procedure and Participants

This study is part of the research project *Physically Active Commuting in Greater Stockholm (PACS)*. Participants, defined as active commuters who walk or cycle to work or study, were recruited through advertisements in Dagens Nyheter (https://www.dn.se/, accessed on 25 May 2004) and Svenska Dagbladet (http://www.svd.se/, accessed on 25 May 2004), two major Stockholm newspapers, between late May and early June 2004. The recruitment period ended in mid-autumn.

**Figure 5:**
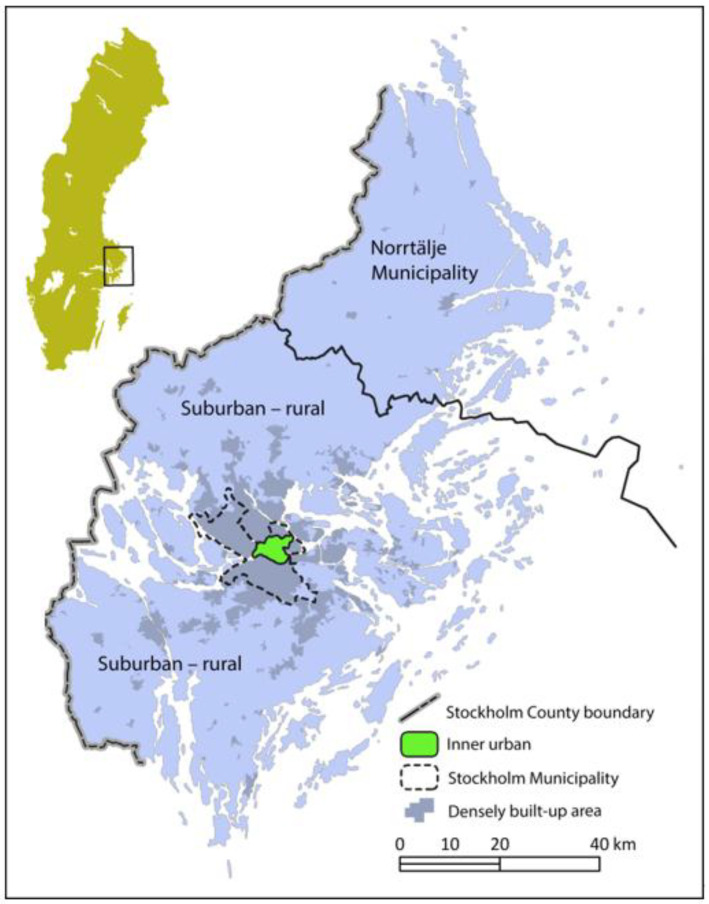
Map over Sweden and Stockholm County, with the suburban–rural study area outside the inner urban area. It will, in the text that follows after this section, be referred to as “Suburban”. The marking for the densely built-up areas displays the conditions in 2010. North is at the top of the image. Figure 5 has previously appeared in three publications (cf. [15,16,17]).

To be eligible, participants had to be at least 20 years old, commute the entire distance to their workplace or educational institution at least once a year, and reside in Stockholm County, excluding the municipality of Norrtälje (see Figure 5).

Based on the information provided in the advertisements about the study, 2148 individuals signed up for the study. A first survey, the Physically Active Commuting in Greater Stockholm Questionnaire (PACS Q1), was distributed in September 2004 (the survey was answered by 94%). At the end of the first questionnaire, participants were asked if they would like to participate in a second survey (PACS Q2). It was distributed in May of the following year (the survey was answered by 92%). Responses were ending in mid-autumn. The respondents walked or cycled in the inner urban or suburban–rural areas of Greater Stockholm, or in both settings. The suburban–rural areas will hereafter be stated as “the suburban areas”.

Advertisement-based recruitment has been compared to on-street recruitment of cycling commuters in terms of their evaluations of route environments [22]. It was hypothesized that individuals recruited in situ would more accurately represent the commuting mode than those recruited through advertisements. Overall, the assessments showed a strong correspondence between the two groups [22].

In the present study, we have exclusively used data from the suburban areas. We asked the participants to evaluate their route environment over a two-week period, and thereafter rate the environmental variables in overall terms. During the studied period, electric cars were scarce or non-existent. Some pedestrians (33.0%) also commuted in the inner urban area; however, only the suburban data were used. It is possible that commuting in one environment affects the ratings of the other. A previous study compared the ratings of cyclists who had commuted in both areas with those who had commuted in only one area [22]. The deviances between the groups were remarkably small.

In this study, 233 participants were included in the analyses (82% women); 41.6% were pedestrians, and 58.4% were dual-mode commuters, i.e., individuals who alternate between walking and cycling. The Ethics Committee North of the Karolinska Institute at the Karolinska Hospital approved the study (Dnr 03-637), and the participants gave their informed consent.

### 2.2. Descriptive Characteristics of the Participants

Descriptive characteristics of the participants were obtained from PACS Q1 and Q2, see Table 1. For characteristics of their walking behaviour, e.g., distance, duration, and frequency of walking trips, see Table A1, Table A2 and Table A3 in Appendix A.

### 2.3. The Physically Active Commuting in Greater Stockholm Questionnaires (PACS Q1 and Q2)

The PACS Q1 and PACS Q2 are self-administered surveys in Swedish, consisting of 35 and 68 items, respectively. The surveys include questions about background factors and various aspects of active commuting. Both surveys are available in the supporting information in Schantz et al. [23].

#### 2.3.1. The Active Commuting Route Environment Scale (ACRES)

To explore the relationships between active transportation and the characteristics of the route environment, the Active Commuting Route Environment Scale (ACRES) was developed [21,22]. The pedestrian version consists of 13 variables. Each variable has two parallel response lines: one for the inner urban area and the other for the suburban areas.

Those responding to the ACRES are asked to rate their overall experience of their self-chosen route environment, based on their active commuting between home and place of work or study, during the last two weeks. The 15-point response scales have adjectival opposites and numbered continuous lines, i.e., numbers from 1 to 15. In addition, number 8, is a neutral option. Thus, three anchors aim to provide additional meaning to the scores along the scale, see Table 2.

### 2.4. Study Area

**Figure 6:**
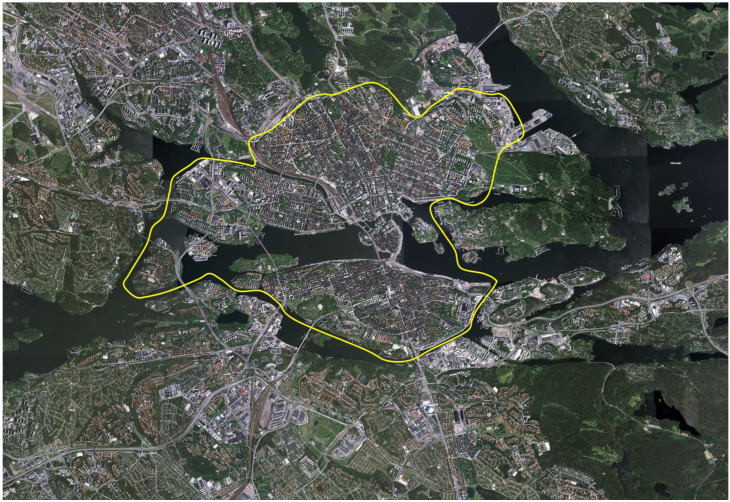
Aerial view of Greater Stockholm. The suburban study area is outside the yellow line. North is at the top of the image. (Copyright:Lantmäteriet, Gävle 2011. Permission 81055230). Figure 6 has previously appeared in three publications (cf. [15,16,17]).

The suburban area surrounds the urban core (see Figure 6) and features a diverse landscape comprising residential neighbourhoods, industrial zones, forests, and agricultural land. Residential areas primarily consist of single-family homes, while multi-story buildings are more common in densely populated districts. Population density generally increases with proximity to underground or commuter train stations.

The main roads often follow historical routes from the agricultural era, unlike the inner urban area, where streets are typically arranged in a grid pattern. Green spaces, including trees and gardens, are interspersed throughout the neighbourhoods, particularly around multi-story housing complexes.

The region is situated within a rift valley landscape, characterized by mostly flat valleys and forests that extend from rural areas toward the city centre. Lakes, the Baltic Sea, and numerous islands further define the geography. Arterial highways traverse the landscape, connecting different parts of the region. More details on the study area can be found in the Statistical Yearbook of Stockholm [24].

### 2.5. Statistical Analyses

Data were entered into the Statistical Package for the Social Sciences (SPSS) and analyzed using version 27.0 (IBM SPSS Inc., Somers, NY, USA). Accuracy checks were performed on PACS Q2 data, including the variables from the ACRES. Only participants with complete ACRES data and background information (*sex*, *age*, *education*, and *income*) were included in the analyses. A significance level of *p* < 0.05 was applied.

Differences in ratings of the variables between men and women were examined with independent *t*-tests.

A visual inspection indicated that the data were approximately normally distributed. Before conducting the analyses, the linearity of environmental variables was assessed visually and found to be reasonably linear.

Multicollinearity was assessed using the variance inflation factor (VIF), with a threshold of >0.80 indicating potential concerns [25] (p. 402). Although some high correlations were observed, the VIF values did not indicate serious multicollinearity issues according to Field [25] (p. 402)).

The threshold for standardized residuals was set at ±3 standard deviations (SD). Some analyses produced residuals exceeding this limit; however, these outliers were included in the analyses, as they were relatively few and relatively close to the threshold. Additionally, Cook’s distance values did not indicate any cause for concern according to Field [25] (p. 383)). A table with the VIF, the standardized residuals, and the Cook’s distance is presented in Appendix A (Table A4).

#### 2.5.1. Background Variables

In our multiple regression and mediation analyses, we included the following background variables: *sex* (binary; coded as 0 for females and 1 for males), *age* (continuous), *education* (binary; 0 for university-level education and 1 for no university-level education), and *income* (categorical; with three groups: 1 for ≤25,000 SEK/month, 2 for 25,001–30,000 SEK/month, and 3 for ≥30,001 SEK/month). Note: SEK refers to Swedish kronor; approximately, 1 € ≈ 9 SEK and 1 US $ ≈ 8 SEK.

#### 2.5.2. Correlation Analyses (CA)

Correlation analyses between the four predictor variables were assessed with Pearson’s correlation coefficient (r). The correlations were r ≤ 0.846.

#### 2.5.3. Multiple Regression Analyses (MRA)

Multiple regression analysis was employed to investigate the relationships between predictor and outcome variables. Results from the linear regression are presented as y-intercepts, unstandardized coefficients (B) with their 95% confidence intervals, and the adjusted R^2^ for the overall models.

#### 2.5.4. Mediation Analyses (MA)

In models where direct or indirect effects were hypothesized, mediation analyses were conducted using the PROCESS macro [26]. Results from these analyses are reported as standardized total, direct, and indirect effects of X on Y. Additionally, the percentage ratio of the standardized indirect effect to the standardized total effect is provided. By default, PROCESS employs 5000 bootstrap samples to compute confidence intervals. Indirect effects were deemed statistically significant if the 95% confidence interval did not include zero.

## 3. Results

### 3.1. Perceptions of the Environmental Variables in Men and Women

Levels of the outcome variables *hinders–stimulates walking* and *unsafe–safe traffic* as well as of the predictor variables *vehicle speed*, *vehicle flow*, *noise*, and *exhaust fumes* are presented in Table 3. A significant difference was noted between men and women regarding *hinders–stimulates walking*; women had a higher mean value.

### 3.2. Correlations Between the Environmental Variables

**Figure 7:**
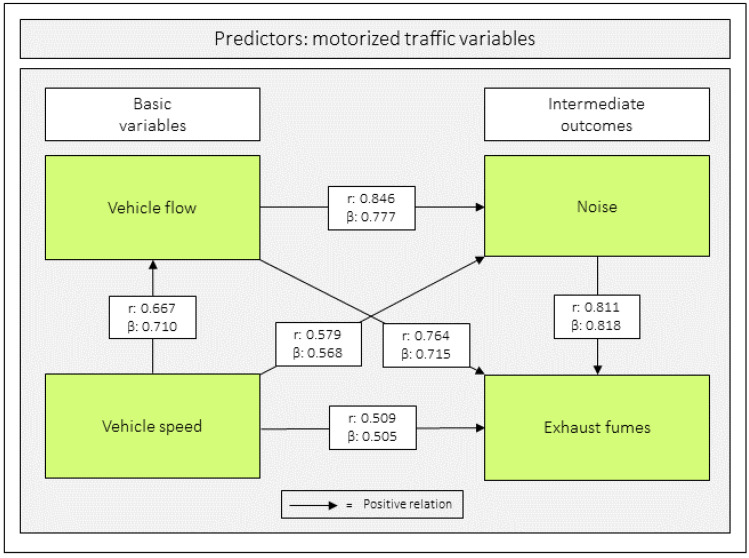
Correlation and regression coefficients between the four predictor variables. Notes: *r* = Pearson’s correlation coefficient and β = unstandardized beta. The figure is based on Table 4 and Table 5.

**Figure A1:**
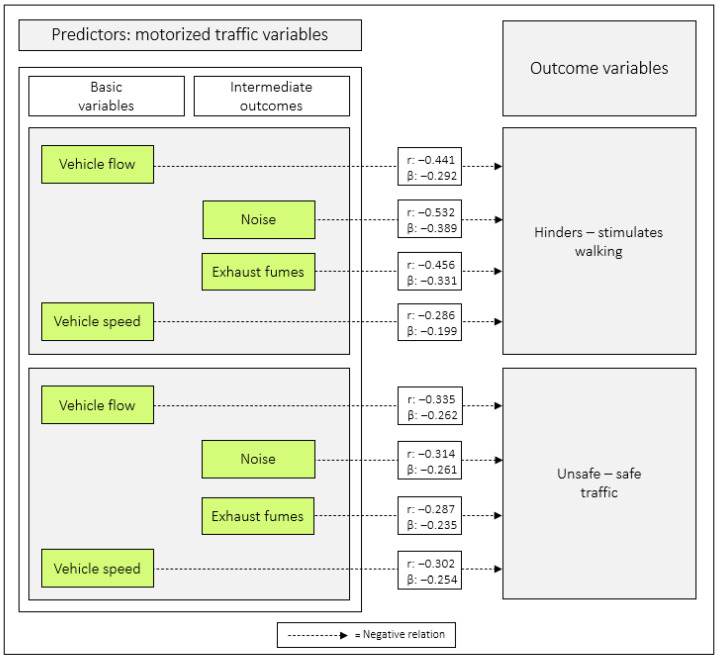
Relations between the individual predictor variables and the outcome variables. Notes: *r* = Pearson’s correlation coefficient and β = unstandardized beta. The figure is based on Table 4, Table A5 and Table A6.

There were positive correlations (r) between all predictor variables (range: 0.509–0.846). In contrast, all predictor variables correlated negatively with the outcome variables *hinders–stimulates walking* (range: −0.286 to −0.532) and *unsafe–safe traffic* (range: −0.287 to −0.335) (Table 4, Figure 7 and Figure A1 in Appendix A).

### 3.3. Relations Between the Predictor Variables

The relations between the predictor variables were analyzed with multiple regression analyses. All regression coefficients were positive (range: 0.505–0.818; *p* < 0.001) (Table 5 and Figure 7).

### 3.4. Relations Between the Basic Variables and the Intermediate Outcomes

**Figure 8:**
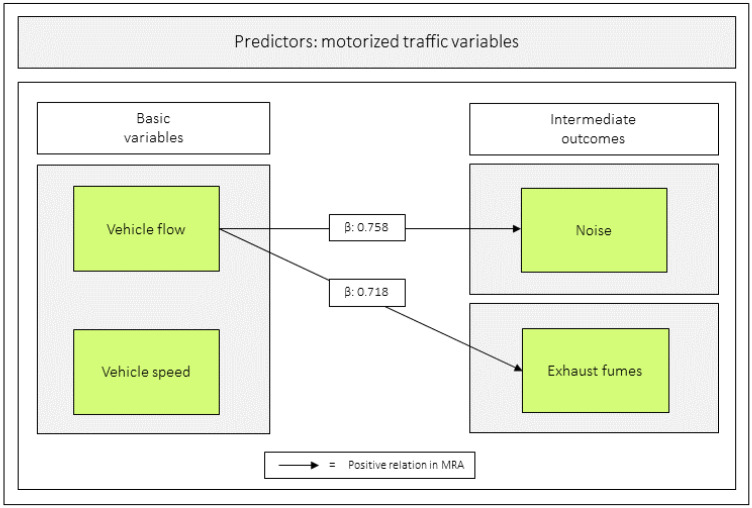
Regression coefficients between *vehicle speed* and *vehicle flow* in relation to *noise* and *exhaust fumes*. Notes: β = unstandardized beta. The figure is based on Table 6.

Of the basic variables *vehicle speed* and *vehicle flow* in relation to the intermediate outcomes *noise* and *exhaust fumes*, only *vehicle flow* was significant. For all values, see Table 6 and Figure 8.

### 3.5. Relations Between Individual Predictor Variables and the Outcome Hinders–Stimulates Walking

All regression coefficients were significant and negative (range: −0.199 to −0.389) with the lowest value for *vehicle speed* and the highest for *noise*. For all the values, see Table A5 and Figure A1.

### 3.6. Relations Between Combinations of Predictor Variables and the Outcome Hinders–Stimulates Walking

**Figure 9:**
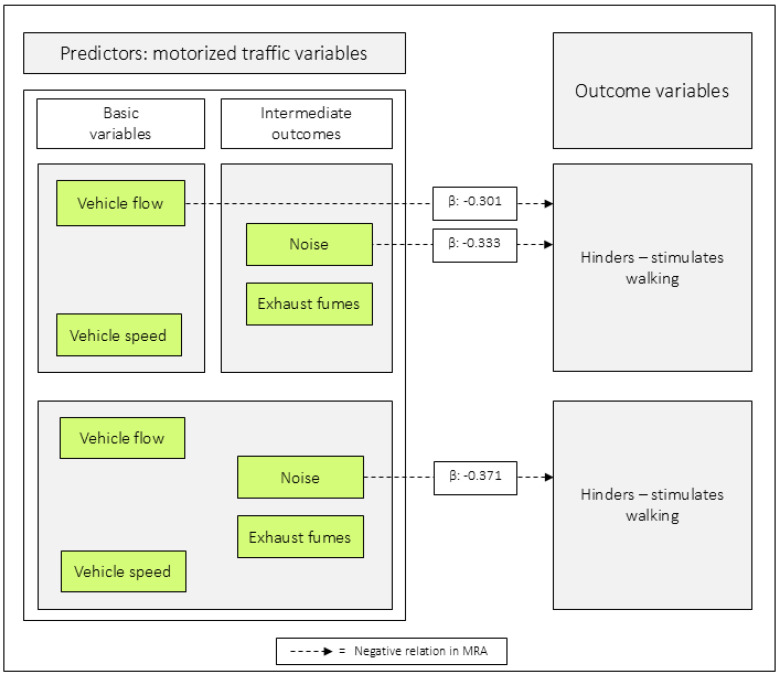
Regression coefficients between combinations of the predictor variables in relation to *hinders–stimulates walking*. Note: β = unstandardized beta. The figure is based on Table 7.

In model 7:1, *vehicle flow* was negatively related to *hinders–stimulates walking*, and in 7:2, *noise* had the equivalent role. When all the motorized traffic variables were included as predictors, only *noise* was negatively related to the outcome (Table 7 and Figure 9).

### 3.7. Relations Between Individual Predictor Variables and the Outcome Unsafe–Safe Traffic

All regression coefficients were significant and negative (range: −0.235 to −0.262), with the lowest value for *exhaust fumes* and the highest for *vehicle flow*. For all values, see Table A6, and Figure A1 in Appendix A.

### 3.8. Relations Between Combinations of Predictor Variables and Unsafe–Safe Traffic as an Outcome

**Figure 10:**
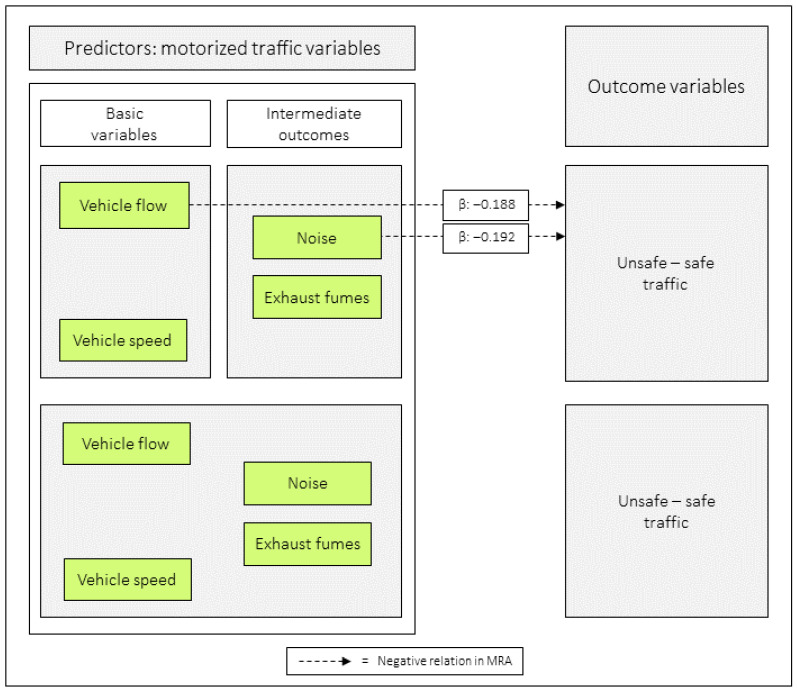
Regression coefficients between combinations of the predictor variables in relation to unsafe–safe traffic. Notes: β = unstandardized beta. The figure is based on Table 8.

**Figure 11:**
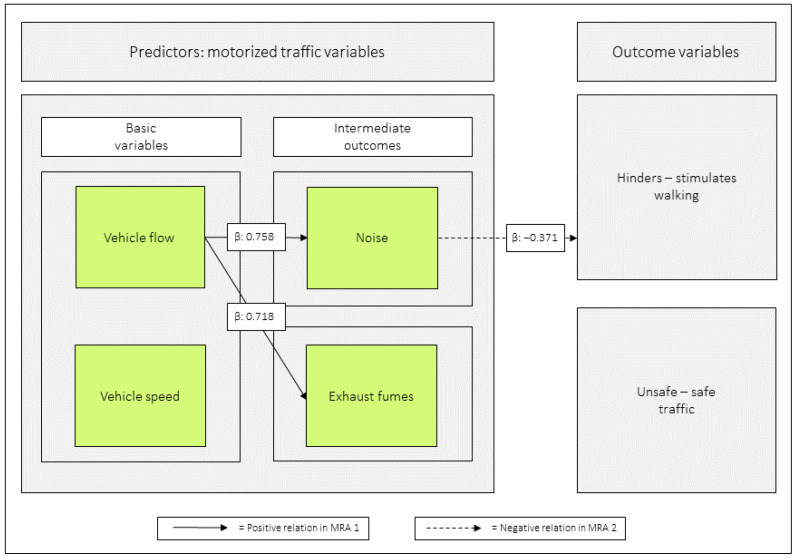
Relations based on multiple regression analysis (MRA) are shown between the predictor variables (MRA 1) and between the predictor variables and the outcome variables (MRA 2). The solid arrows, based on Table 6, illustrate that when the basic variables are analyzed jointly as predictors, *vehicle flow* is positively related to both intermediate outcomes. The dashed arrow, based on Table 7, shows that when all motorized traffic variables are analyzed together as predictors, only *noise* is negatively related to *hinders–stimulates walking*. β = unstandardized beta.

In model 8:1, *vehicle flow* was negatively related to *unsafe–safe traffic*, and in 8:2, *noise* had the equivalent role. When all the motorized traffic variables were included as predictors, none was significantly related to the outcome (Table 8 and Figure 10 and Figure 11).

### 3.9. Mediation

Mediation analyses were conducted in models where a potential mediating effect was of interest (Table 9). In addition to the previously analyzed variables, a composite variable was created by multiplying *vehicle flow* by *vehicle speed*, as this combination possibly could influence the outcome variables. Only mediated effects (*p* < 0.05) corresponding to 40% or more are commented upon. 40% was chosen since it represents a sizable effect.

An indirect effect of *vehicle speed* on *noise* is mediated by *vehicle flow* (95%), and an indirect effect of *vehicle speed* on *exhaust fumes* is mediated by *vehicle flow* (101%). *Noise* mediates the effect of *vehicle flow, vehicle speed,* and the composite variable to *hinders–stimulates walking* (the indirect effects were 111%, 116% and 126%, respectively).

### 3.10. A Graphic Illustration of Significant Relations Based on the Commuting Pedestrians’ Perceptions and Appraisals of Their Route Environments

**Figure 12:**
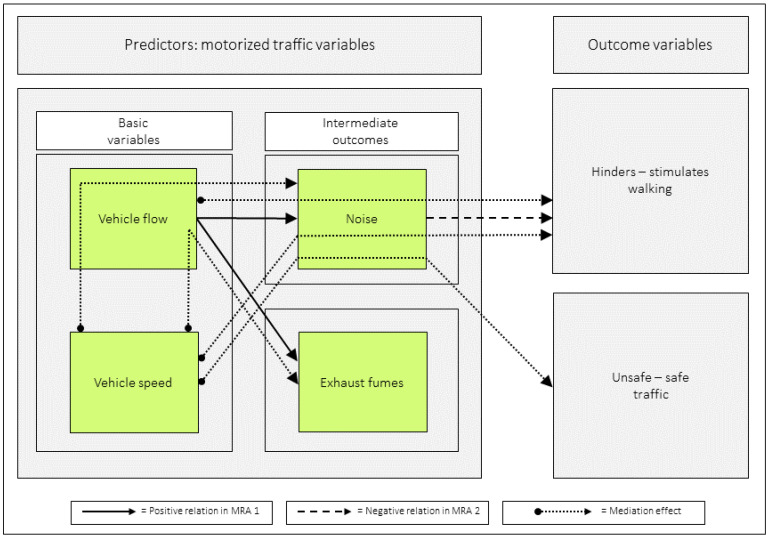
The solid arrows are derived from the multiple regression analyses in Table 6 (MRA 1). These arrows show that when *vehicle speed* and *vehicle flow* are analyzed together as predictors, *vehicle flow* is related to both intermediate outcomes. The dashed arrow comes from the multiple regression analyses in Table 7 (MRA 2) and indicates that when all motorized traffic variables are considered as predictors, *noise* relates negatively to *hinders–stimulates walking*. The dotted arrows are based on mediation analyses in Table 9. These arrows illustrate that *vehicle speed* indirectly impacts *noise* and *exhaust fumes* through *vehicle flow,* and also influences both outcome variables through *noise*. Additionally, *vehicle flow* indirectly affects *hinders–stimulates walking* via *noise*. Figure 12 has previously appeared in Andersson [15].

When adding significant indirect effects corresponding to 40% or more to Figure 11, and removing numerical data, the following graphic emerges, see Figure 12 (MA 9:9 not included). Note that we included MA 9:8 even if it did not reach 40% (the effect was 39%).

## 4. Discussion

This is, to our knowledge, the first time that perceptions and relations between four variables connected to motorized vehicles and outcomes in terms of hindering–stimulating walking as well as unsafety–safety for reasons of traffic, are studied in suburban and rural settings using correlational, multiple regression, and mediation analyses with the aim of establishing relations between these variables.

One of the primary results regarding the perceptions of four motorized traffic variables was that *noise* was the primary negative predictor variable in relation to *hinders–stimulates walking*. The regression equation was y = 12.2 − 0.37 noise (all *p*-values ≤ 0.001, Adj. R^2^ = 0.29).

Concerning the other outcome, *unsafe–safe traffic*, it was more difficult to identify a primary predictor variable, as none of the motorized traffic variables were significant when jointly analyzed in multiple regression analysis. *Vehicle speed* was the closest one (*p* = 0.095), thus suggesting a potential effect.

The mediation analyses showed that (1) *vehicle speed* had an indirect effect on *unsafe–safe traffic*, via *noise*, (2) both *vehicle speed* and *vehicle flow* had, via *noise*, indirect effects on *hinders–stimulates walking*, and (3) *vehicle speed* had, via *vehicle flow*, an indirect effect on *noise* and *exhaust fumes*.

How these and other relations can be further analyzed will be discussed below. In doing so, we will integrate the results from correlation (CA), multiple regression (MRA), and mediation (MA) analyses.

We have previously studied how motorized traffic variables are perceived by pedestrians in the inner urban area of Stockholm [16]. For a comparison between perceptions in the two settings, see Table 10.

Table 10 displays levels and ratios between perceptions of the motorized traffic variables in the two environments. The ratios of the mean levels of the inner urban/suburban ratings of *vehicle speed, vehicle flow, noise,* and *exhaust fumes* are consistently above 1.0. This finding aligns with previous studies on cycle commuters in the same settings (see Table 11 [18,19]).

### 4.1. The Relationships Between Perceptions of the Predictor Variables of Motorized Traffic

A few high correlations were identified between the predictor variables of motor traffic, e.g., between *noise* and *vehicle flow* (*r* = 0.85) followed by between *noise* and *exhaust fumes* (*r* = 0.81). High correlations can be problematical when trying to pinpoint the role of a specific variable in relation to an outcome, e.g., *hinders–stimulates walking* and *unsafe–safe traffic*. One way of avoiding this is to include more participants; another is to analyze the relationships between the traffic and outcome variables gradually, with different statistical tools. We have chosen the latter strategy.

### 4.2. Vehicle Speed and Vehicle Flow in Relation to Noise and Exhaust Fumes

*Vehicle speed* and *vehicle flow* were related, as indicated by a correlation coefficient of *r* = 0.67. Both *vehicle speed* and *vehicle flow* were individually related to *noise* in MRA. When jointly analyzed, only *vehicle flow* was related to *noise* and *exhaust fumes*. The MA demonstrated that *vehicle speed* had indirect effects, via *vehicle flow*, on both *noise* (95%) and *exhaust fumes* (101%). The basic variable *vehicle speed* protrudes, therefore, as an important origin to both of the intermediate outcomes *noise* and *exhaust fumes*.

Our separate analysis of *vehicle speed* and *vehicle flow* in relation to *noise* in the inner urban area compared to the suburban area indicated, via the regression coefficients, that vehicle speed had a similar impact on noise in the two settings, whereas vehicle flow had a greater impact on noise in the suburban area. 

Although the MRA showed no relationship between *exhaust fumes* and either outcome, it is worth noting that pedestrians and others in urban environments are often exposed to unseen and scentless pollutants, such as particulate matter (PM).

### 4.3. The Motorized Traffic Variables in Relation to the Outcome Variable Hinders–Stimulates Walking

Individually, each motorized traffic variable was negatively related to *hinders–stimulates walking.* This suggests that if only a few motor traffic variables are included as indicators of motorized traffic in studies aimed at evaluating their effect, the potentially more fundamental relationships will remain undisclosed.

When combining the basic variables *vehicle speed* and *vehicle flow*, and analyzing them in MRA, with *hinders–stimulates walking* as the outcome, only *vehicle flow* was negatively related. When combining the intermediate outcomes *noise* and *exhaust fumes*, *noise* had the corresponding role. When all four motorized traffic variables were included as predictors, only *noise* was negatively related to *hinders–stimulates walking* (see Figure 9).

**Figure 13:**
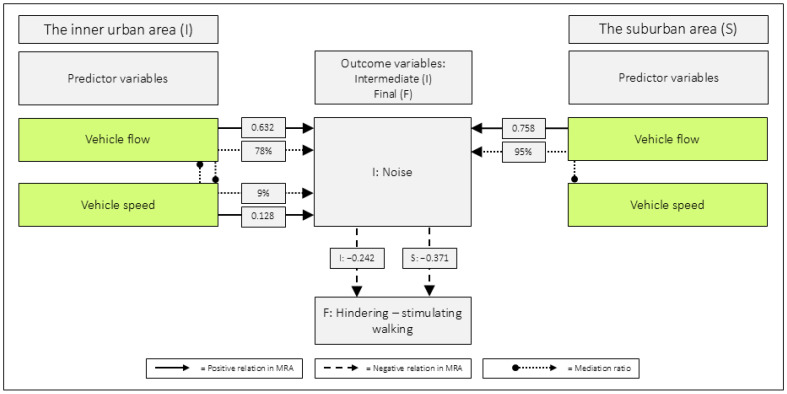
The background to the perceptions of noise. Data for the inner urban area is from Andersson et al. [16], while data for the suburban area is from the present study. MRA = Multiple Regression Analysis (values represent unstandardized B). The mediation ratio indicates the percentage of the standardized indirect effect relative to the standardized total effect. Figure 13 has previously appeared in Andersson [15].

Perceptions of *noise* are influenced by both *vehicle flow* and *vehicle speed*. In the inner urban area, MRA revealed that both variables were positively related to *noise* [16]. MA further revealed that *vehicle flow* mediated the effect of *vehicle speed* on *noise*, and vice versa. In suburban areas, however, only *vehicle flow* was directly related to *noise* in MRA. The MA demonstrated that *vehicle speed* had an indirect effect on *noise* through *vehicle flow* (see Figure 13).

The unstandardized B regarding *noise*, in relation to *hinders–stimulates walking* in the inner urban area was −0.242 (95% CI: −0.415 to −0.070) [16]. The corresponding value in the suburban areas was −0.371 (95% CI: −0.555 to −0.186). Although not significantly higher, due to overlapping CI, it is interesting that the values diverged in that direction. This is, not least, the case, given that the mean level of noise was perceived as about 50% lower in suburbia (see Table 10).

A reasonable explanation for this is that individuals might be more sensitive to *noise* in a suburban setting. Thus, this may load the variable with a more negative impact in suburbia, compared to in an inner urban context.

However, suburban areas are generally greener than inner urban areas, and a greener environment has been reported to correspond with lower noise annoyance [28,29]. In line with that, it is not anticipated that the regression coefficients diverge in that direction. Thus, this issue deserves future attention.

The experience of noise as a source of discomfort is not unique to the study area; it has been reported as a deterrent to pedestrians in various countries and settings. For example, a U.S. study on recreational walking examined the influence of environmental variables, time, and distance on walking preferences, finding that participants preferred low-noise environments over high-noise ones [30]. Similarly, a German study on pedestrian commuters, employing a mixed-methods approach, found that noise levels negatively affected commuting experiences [31]. In Spain, a qualitative study on the role of the built environment in short walking trips for transportation identified traffic noise as an unpleasant factor that could influence route choices [32]. Furthermore, a Chilean study based on walk-along interviews reported that negative walking experiences increased as participants approached main streets with high motorized traffic and noise [5].

While barriers can help reduce road traffic noise annoyance and improve overall sound quality [33], noise remains a significant threat to both health and environmental well-being. Its harmful effects include elevated stress hormone levels, increased blood pressure, and sleep disturbances, which in turn raise the risk of cardiovascular diseases and metabolic disorders such as ischemic heart disease, obesity, and type 2 diabetes [34,35,36,37]. Noise is often referred to as a “silent killer”, and as Münzel et al. [35] (p. 831) explain, “… noise may exert its effects either directly, through synaptic interactions or indirectly, through the emotional and the cognitive perception of sound.”

Recognizing these risks, the World Health Organization (WHO) recommends that road traffic noise should not exceed 53 dB Lden, as higher levels are associated with adverse health effects [36] (p. 30). In Stockholm County, however, 30% of adult residents are exposed to traffic noise exceeding this threshold at the façades of their homes [38] (p. 15).

Interestingly, long-term exposure to transportation noise has also been associated with reduced physical activity levels [39]. This may be due to the “barrier effect” of traffic, which has been observed when major roads separate residential areas from and green spaces [40]. Furthermore, walking has been reported to be hindered when both traffic volume and speed are rated as high [41]. This finding aligns with another study, which suggests that high traffic volume, in combination with high traffic speeds, may hinder physical activity [42]. However, given the results from the present study, the actual problem might be *noise*, a variable that was not included in any of the two studies mentioned.

A common recommendation for reducing traffic noise is to replace traditional cars with electric vehicles. However, this shift will only lead to modest reductions in road traffic noise. Research suggests that there is a threshold around 30 km/h [43,44] (p. 3, p. 4). Below this speed, electric vehicles are quieter than those with internal combustion engines. Above 30 km/h, which is frequent in many urban, suburban, and rural areas, tyre friction becomes the dominant source of noise.

### 4.4. Comments on Relations Between Noise and Vehicle Flow

Although vehicle flow is highly correlated with noise, with a correlation coefficient of *r* = 0.85, there are reasons to emphasize that our perception of a certain *vehicle flow* can vary. It may vary considerably depending on its back- and foreground features, the proximity, visibility, and the direction of the *vehicle flow* in terms of moving towards or away from a pedestrian (cf. Figure 3a,b as well as Figure 4a,b). As a result, the impact of a given *vehicle flow* can differ depending on several issues. In contrast, *noise* is more omnipresent in urban or suburban–rural environments and can reach us almost regardless of the visual conditions coupled to the flow.

Studies of commuting cyclists in the suburban and rural areas of Stockholm, Sweden, have shown that both *vehicle flow* and *noise* can be negatively related to *inhibiting–stimulating* cycling [18]. The pedestrians rated, on average, about 10% lower levels of *vehicle flow* and 5% lower levels of noise compared to cyclists [18], but this may not explain why *vehicle flow* did not emerge as a significant and independent negative predictor. It is more likely that it was due to the considerably greater statistical power in the cyclist group with 1098 cyclists compared to 233 pedestrians in the suburban–rural areas.

It is essential to explore methods that can facilitate these types of studies without requiring large groups of respondents. Therefore, we will revisit these perspectives in the final part of this Discussion, under the heading “4.7. Exploring Measurement Perspectives”.

### 4.5. The Motorized Traffic Variables in Relation to the Outcome Variable Unsafe–Safe Traffic

Individually, each variable was negatively related to *unsafe–safe traffic*. As mentioned, this indicates that if few or imprecisely defined motor traffic variables are included as indicators for motorized traffic in studies aiming to evaluate their effect, the fundamental relations might remain undisclosed.

When combining the basic variables *of vehicle speed and vehicle flow and analyzing them in MRA, with unsafe–safe traffic as the* outcome, *vehicle flow* was negatively related to the outcome. When combining the intermediate outcomes *noise* and *exhaust fumes*, *noise* had the corresponding role. Finally, when all motorized traffic variables were included as predictors, none of them were found to be significantly related to the outcome. However, *vehicle speed* had a tendency (*p* = 0.095) to have such an effect. The MA demonstrated that *vehicle speed,* via *noise,* had an indirect effect on *unsafe–safe traffic* (the indirect effect was 39%).

Since safety has been discussed as an important issue for pedestrians [45,46,47], it is surprising that none of the motorized traffic variables were significantly related to unsafe versus *safe traffic* in MRA. This may be due to multicollinearity between the predictors of motor traffic, such as noise and vehicle flow, which are highly correlated (*r* = 0.85).

Nonetheless, with respect to road safety, speed management should be a top priority. In accordance with that, a campaign initiated by the United Nations (UN) has been launched to limit the speed in urban areas to 30 km/h [4] (p. 60). This initiative will not only save lives directly but also contribute to a reduction in noise levels. Over time, lower noise pollution is associated with improved public health.

### 4.6. External Validity in Relation to Different Subgroups in a Population

In a study examining how cyclists of different experience levels (regular, frequent, occasional) and potential cyclists evaluated various routes in relation to their likelihood of usage, the order of preferences was consistent across all groups [48]. This means that the differences in preferences for various route environmental features were similar across all cyclist groups and potential cyclist groups. However, the sensitivity to usage of each type of route environment differed between the groups, with potential cyclists being the most selective of the route environments, followed by those in the other groups, in the order of occasional, frequent, and regular cyclists. In relation to the present study, it suggests that potential pedestrians may be more sensitive to, for example, noise levels, than those who walk on a regular basis. Thus, high standards in this and other environmental respects need to be taken into consideration from a public health perspective.

### 4.7. Exploring Measurement Perspectives

In the Introduction, we suggested that the perceptions and appraisals coupled to objective levels of the four traffic variables may differ between inner urban and suburban –rural areas. We reason that in urban centres, large buildings may obscure or reduce the prominence of certain traffic variables, such as our perception of *vehicle flow*, compared to suburban or rural settings. Also, parked cars can hinder the visibility of traffic flows. Furthermore, higher buildings can create canyon effects, concentrating the levels of, e.g., exhaust fumes.

Contrary to what was found among commuting cyclists [18], we did not note that both *vehicle flow* and *noise* were negative predictors among pedestrians in suburban–rural areas, and have indicated that it may be due to the differences in statistical power between the studies.

**Figure 14:**
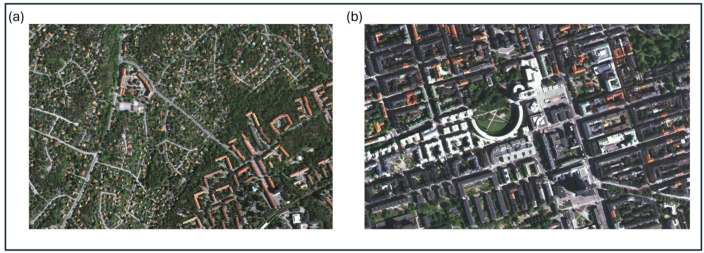
(**a**,**b**) An illustration of a common form of a suburban area (Figure 14a) and a typical inner urban area (Figure 14b) in Greater Stockholm, Sweden. In the suburban area, the directions of the roads shift relatively frequently, whereas in the inner urban area, the streets are laid out in long, linear patterns. This difference affects both the visibility of traffic flow and the speed of traffic, as well as the spatial distribution of traffic-related noise and exhaust emissions. Consequently, studies on the perception of individual motorized traffic variables call for further investigation within and between clearly defined local contexts.

We have clustered suburban and rural settings, which also include national highways with high levels of traffic flow and speed. Given the complexity of these settings, they warrant further exploration. In this respect, isolating and controlling for the environmental features under study is likely to be a fruitful approach. Future studies would also likely benefit from both separate and comparative investigations of typical suburban and rural areas, respectively. In the present study, we compare findings from the suburban and the inner urban areas. Therefore, we here illustrate a common form of a suburban area (Figure 14a) and a typical inner urban area (Figure 14b) in Greater Stockholm, Sweden. In the figure legend, we comment on the conceivable consequences of the striking differences in these built-up areas (Figure 14a,b).

**Figure 15:**
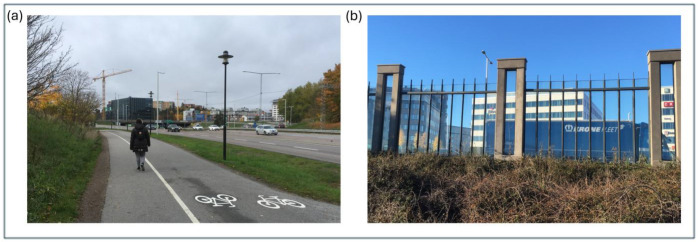
(**a**,**b**) The national highway settings at Bergshamraleden (Highway E18 at Järva Krog), and at Uppsalavägen (Highway E4 at the northern part of Brunnsviken). Figure 14a illustrates the most frequent types of path settings along national highways for pedestrians and bicyclists, whereas Figure 14b shows an attempt to reduce traffic noise for pedestrians and cyclists along Uppsalavägen using transparent shields (Photo: Peter Schantz).

Another pathway is to create experimental study conditions in which only one variable is modified. That would probably facilitate a more in-depth understanding of these phenomena. Let us consider such a possibility presented in Figure 15a,b. In the suburban–rural area, several national highways pass by. Along many of these routes, both pedestrians and cyclists are forced to travel without any protection from noise, as no alternative paths are available (Figure 15a).

However, there are some settings (cf. Figure 15b) that would facilitate experimental studies of the effect of visually perceiving a traffic flow or not, while the auditory exposure remains constant. In that way, the impact of observing a vehicle flow can be easily studied.

The ratings used in this study represent mean values for the individual routes taken by pedestrians to work. They represent distinct values by indicating both the mean and the minimum levels of route environmental variables that most likely need to be reached in order to attract greater levels of cycling and walking.

**Figure 16:**
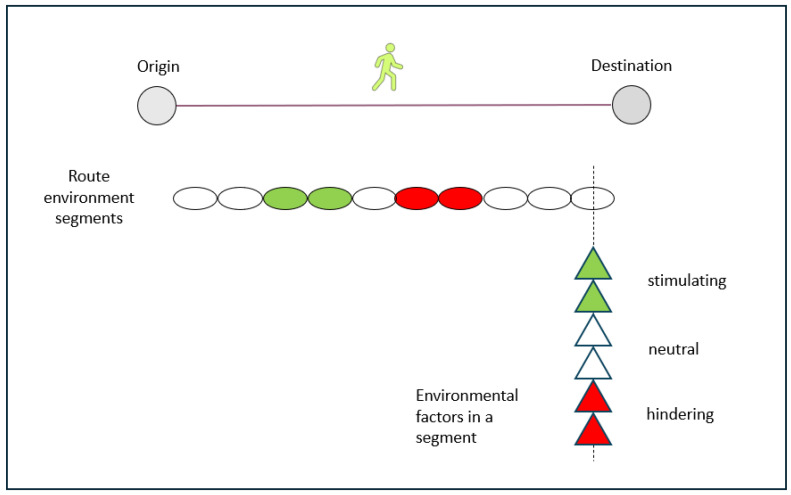
A key conceptual illustration showing how the composition and magnitude of single route environment variables influence each route environment segment in terms of making it overall neutral, dominantly stimulating, or dominantly hindering, respectively, for walking.

However, often the environment changes along the route, and from teaching experiences with students, it is clear that even limited aspects of route settings can be negative enough to deter walking and cycling behaviours. The fact that route environments often differ between the origin and the destination can be used for research purposes. The conceptual illustration below is intended to stimulate the development of research strategies for these purposes (Figure 16). From the origin to the destination of a walking or cycling trip, one passes through several route segments, which are defined as discrete spatial units with the same composition of environmental variables and levels. Depending on the composition of environmental variables that act within the segments, the overall ratings of them can be, for example, stimulating, neutral, or hindering for walking or cycling. Through studying these matters by both quantitative and qualitative measures, one can complement other methods. Each segment can be studied in relation to how the environmental variables affect both the intermediate and the final outcomes in the model presented in Figure 1.

## 5. Conclusions

In suburban–rural route environments of a metropolitan setting, *noise* related negatively to the outcome *hinders–stimulates walking*. With respect to the other outcome, *unsafe–safe traffic*, none of the four traffic variables were significantly related when jointly analyzed in MRA. *Vehicle speed* had, however, a certain tendency. The MA revealed that vehicle speed, through vehicle flow, exacerbates *noise* and has indirect negative effects on both outcomes. Hence, if levels of *vehicle speed* are reduced, *noise* levels will decrease, and the walking experience is likely to be enhanced, positively influencing the amount of physical activity.

This study can advance the understanding of how results from other studies, which may have used fewer traffic variables in an effort to highlight the influence of the traffic environment, can be interpreted. This understanding can assist research, transportation, and planning societies in selecting relevant proxy variables.

Finally, we have critically explored the current measurement conditions and suggested possible future pathways, including experimental studies, that we believe will enhance opportunities to explore and detect even smaller effects of motorized traffic variables, and enable fewer pedestrians and bicyclists to be involved.

## Figures and Tables

**Table 1:** Descriptive characteristics of participants (*n* = 228–233) *.

Descriptive Characteristics of the Participants		
Females **, %		82
Age in years **, mean ± SD		50.0 ± 9.8
Weight in kg, mean ± SD		68.4 ± 10.4
Height in cm, mean ± SD		169.9 ± 7.8
Body mass index, mean ± SD		23.7 ± 3.0
Gainful employment, %		97
Educated at university level **, %		70
Income **:	≤25,000 SEK *** a month, %	53
	25,001–30,000 SEK *** a month, %	28
	≥30,001 SEK *** a month, %	18
Participant and both parents born in Sweden, %		83
Having a driver’s licence, %		88
Usually access to a car, %		70
Leaving home 7–9 a.m. to walk to work or study, %		70
Leaving place of work or study 4–6 p.m. to walk home, %		69
Number of walking-commuting trips per year ****, mean ± SD		226 ± 159
Overall physical health either good or very good, %		72
Overall mental health either good or very good, %		82

Notes: * 233 individuals have complete data regarding ACRES and the four background variables that are used in the analyses; ** Included in the multiple regression analyses and in the mediation analyses; *** SEK = Swedish crowns (SEK): €1 ≈ 9 SEK; US$1 ≈ 8 SEK; **** Among the 165 participants, annual walking-commuting trips ranged from 6.50 to 546. Five extreme values were replaced using simple mean imputation based on the remaining 160 cases, preserving the sample size while reducing variance. Missing data in at least one of the 12 months led to exclusion from the sum score. This variable was used only for the descriptive statistics. The low response rate is due to missing values in at least one of the 12 months, leading to exclusion from the sum score.

**Table 2:** The applied predictor and outcome variables.

Variable	Ratings and Verbal Anchors			Variable Name
	1	8	15	
How do you find the flow of motor vehicles (number of cars) along your route?	Very low	Neither low nor high	Very high	Flow of motor vehicles **
How do you find the speeds of motor vehicles (taxis, lorries, ordinary cars, buses) along your route?	Very low	Neither low nor high	Very high	Speeds of motor vehicles ***
How do you find the noise levels along your route?	Very low	Neither low nor high	Very high	Noise
How do you find the exhaust fume levels along your route?	Very low	Neither low nor high	Very high	Exhaust fumes
Do you think that, on the whole, the environment you walk in stimulates/hinders your commuting?	Hinders a lot	Neither hinders nor stimulates	Stimulates a lot	Hinders–stimulates walking *
How unsafe/safe do you feel in traffic as a pedestrian along your route?	Very unsafe	Neither unsafe nor safe	Very safe	Unsafe–safe traffic *

Notes: This is a translation of the original ACRES in Swedish. * Outcome variable. ** Sometimes the truncated term vehicle flow is used as a synonym. *** Sometimes, the truncated term vehicle speed is used as a synonym.

**Table 3:** Levels of perceptions and appraisals of the environmental variables in males and females (mean, SD, and (95% CI)).

	Outcome Variables		Predictor Variables			
	Hinders–Stimulates Walking *	Unsafe–Safe Traffic	Vehicle Speed	Vehicle Flow	Noise	Exhaust Fumes
Men (*n* = 43)	10.3	12.0	8.37	7.05	7.09	6.60
	3.30	3.09	3.72	3.81	3.57	3.42
	(9.26–11.3)	(11.0–12.9)	(7.23–9.52)	(5.87–8.22)	(5.99–8.19)	(5.55–7.66)
Women (*n* =190)	11.4	12.2	7.75	6.66	6.54	6.49
	2.99	3.22	3.81	4.29	4.01	4.03
	(11.0–11.9)	(11.7–12.7)	(7.20–8.29)	(6.04–7.27)	(5.97–7.12)	(5.92–7.07)

Notes: For an explanation of the scale, see Section 2.3.1. * Significant difference (*p* < 0.05).

**Table 4:** Correlation matrix for the environmental variables (*r*).

	Hinders–Stimulates Walking	Unsafe–Safe Traffic	Vehicle Speed	Vehicle Flow	Noise	Exhaust Fumes
Hinders–stimulates walking	–					
Unsafe–safe traffic	0.264 *	–				
Vehicle speed	−0.286 *	−0.302 *	–			
Vehicle flow	−0.441 *	−0.335 *	0.667 *	–		
Noise	−0.532 *	−0.314 *	0.579 *	0.846 *	–	
Exhaust fumes	−0.456 *	−0.287 *	0.509 *	0.764 *	0.811 *	–

Note: * Pearson’s correlation coefficient (r) is significant at the 0.01 level.

**Table 5:** Relations between the predictor variables.

Model	Outcome	y-Intercept	*p*-Value	Predictor	Unstandardized B	*p*-Value	Adj. R^2^
		(95% CI)			(95% CI)		
5:1	Noise	5.22	<0.001	Vehicle speed	0.568	<0.001	0.366
		(2.61–7.84)			(0.460–0.677)		
5:2	Noise	2.16	0.017	Vehicle flow	0.777	<0.001	0.713
		(0.393–3.92)			(0.709–0.844)		
5:3	Exhaust fumes	5.14	<0.001	Vehicle speed	0.505	<0.001	0.263
		(2.33–7.95)			(0.389–0.622)		
5:4	Exhaust fumes	2.12	0.052	Vehicle flow	0.715	<0.001	0.577
		(−0.017–4.25)			(0.633–0.797)		
5:5	Exhaust fumes	1.30	0.187	Noise	0.818	<0.001	0.654
		(−0.637–3.25)			(0.739–0.898)		
5:6	Vehicle flow	4.19	0.001	Vehicle speed	0.710	<0.001	0.469
		(1.64–6.74)			(0.604–0.816)		

Notes: The background variables *sex*, *age*, *education*, and *income* were included in the analysis. Only significant (*p* < 0.05) background variables are reported. The background variable *age* was significant in model 5:1: unstandardized B = −0.080 (*p* ≤ 0.001), model 5:3: unstandardized B = −0.056 (*p* = 0.016) and in model 5:6: unstandardized B = −0.076 (*p* ≤ 0.001).

**Table 6:** Relations between the predictor variables *vehicle speed* and *vehicle flow* and the intermediate outcomes *noise* and *exhaust fumes*.

Model	Intermediate Outcome	y-Intercept (95% CI)	*p*-Value	Predictor	Unstandardized B (95% CI)	*p*-Value	Adj. R^2^
6:1	Noise	2.05 (0.25–3.85)	0.026	Vehicle speed	0.031 (−0.067–0.128)	0.536	0.712
				Vehicle flow	0.758 (0.668–0.848)	<0.001	
6:2	Exhaust fumes	2.13 (−0.05–4.32)	0.055	Vehicle speed	−0.004 (−0.122–0.114)	0.945	0.575
				Vehicle flow	0.718 (0.608–0.827)	<0.001	

Notes: The background variables *sex*, *age*, *education*, and *income* were included in the analysis. Only significant (*p* < 0.05) background variables are reported. There were no significant background variables.

**Table 7:** Relations between combinations of predictor variables and the outcome *hinders–stimulates walking*.

Model	Outcome	y-Intercept (95% CI)	*p*-Value	Predictor	Unstandardized B (95% CI)	*p*-Value	Adj. R^2^
7:1	Hinders–stimulates walking	11.3 (8.98–13.7)	<0.001	Vehicle speed	0.014 (−0.112–0.140)	0.822	0.208
				Vehicle flow	−0.301 (−0.418 to −0.184)	<0.001	
7:2	Hinders–stimulates walking	12.4 (10.2–14.6)	<0.001	Noise	−0.333 (−0.483 to −0.183)	<0.001	0.292
				Exhaust fumes	−0.068 (−0.215–0.079)	0.361	
7:3	Hinders–stimulates walking	12.2 (10.0–14.5)	<0.001	Vehicle speed	0.025 (−0.094–0.145)	0.677	0.287
				Vehicle flow	0.037 (−0.133–0.207)	0.668	
				Noise	−0.371 (−0.555 to −0.186)	<0.001	
				Exhaust fumes	−0.080 (−0.232–0.072)	0.301	

Notes: The background variables *sex*, *age*, *education*, and *income* were included in the analysis. Only significant (*p* < 0.05) background variables are reported. *Sex* was significant in all models: 7:1: unstandardized B = −1.060 (*p* = 0.027); 7:2: unstandardized B = −0.981 (*p* = 0.030); 7:3: unstandardized B = −0.991 (*p* = 0.029). *Age* was significant in model 7:1: unstandardized B = 0.042 (*p* = 0.031). Table 7 has previously appeared in Andersson [15].

**Table 8:** Relations between combinations of predictor variables and *unsafe–safe traffic* as an outcome.

Model	Outcome	y-Intercept (95% CI)	*p*-Value	Predictor	Unstandardized B (95% CI)	*p*-Value	Adj. R^2^
8:1	Unsafe–safe traffic	15.4 (12.8–18.0)	<0.001	Vehicle speed	−0.120 (−0.260–0.019)	0.089	0.104
				Vehicle flow	−0.188 (−0.317 to −0.059	0.005	
8:2	Unsafe–safe traffic	14.9 (12.4–17.5)	<0.001	Noise	−0.192 (−0.369 to −0.015)	0.033	0.082
				Exhaust fumes	−0.084 (−0.258–0.090)	0.343	
8:3	Unsafe–safe traffic	15.6 (13.0–18.2)	<0.001	Vehicle speed	−0.119 (−0.259–0.021)	0.095	0.100
				Vehicle flow	−0.113 (−0.312–0.086)	0.264	
				Noise	−0.058 (−0.273–0.157)	0.596	
				Exhaust fumes	−0.043 (−0.221–0.135)	0.634	

Notes: The background variables *sex*, *age*, *education*, and *income* were included in the analysis. There were no significant background variables. Table 8 has previously appeared in Andersson [15].

**Table 9:** Mediation analyses between the four predictor variables of motor traffic (MA 9:1–MA 9:4), between the same predictor variables and the two outcome variables (MA 9:5–MA 9:8), as well as between the composite variable (*vehicle flow × vehicle speed*) and the outcome variables (MA 9:9–MA 9:10).

Model	Predictor (X)	Mediator (M)	Outcome (Y)	Standardized Total Effect of X on Y		Standardized Direct Effect of X on Y		Standardized Indirect Effect of X on Y		
				b	*p*-Value	b	*p*-Value	b	95% CI	% of Total Effect *
MA 9:1	Vehicle flow	Vehicle speed	Noise	0.829	<0.001	0.809	<0.001	0.020	−0.050–0.087	2
MA 9:2	Vehicle speed	Vehicle flow	Noise	0.548	<0.001	0.029	0.536	0.518	0.433–0.605	95
MA 9:3	Vehicle flow	Vehicle speed	Exhaust fumes	0.766	<0.001	0.768	<0.001	−0.003	−0.084–0.082	0
MA 9:4	Vehicle speed	Vehicle flow	Exhaust fumes	0.489	<0.001	−0.004	0.945	0.493	0.400–0.587	101
MA 9:5	Vehicle flow	Noise	Hinders–stimulates walking	−0.399	<0.001	0.042	0.687	−0.441	−0.609 to −0.288	111
MA 9:6	Vehicle speed	Noise	Hinders–stimulates walking	−0.246	<0.001	0.039	0.566	−0.285	−0.379 to −0.195	116
MA 9:7	Vehicle flow	Noise	Unsafe–safe traffic	−0.344	<0.001	−0.252	0.033	−0.092	−0.274–0.082	27
MA 9:8	Vehicle speed	Noise	Unsafe–safe traffic	−0.301	<0.001	−0.184	0.017	−0.117	−0.224 to −0.028	39
MA 9:9	Composite variable	Noise	Hinders–stimulates walking	−0.349	<0.001	0.089	0.330	−0.438	−0.595 to −0.298	126
MA 9:10	Composite variable	Noise	Unsafe–safe traffic	−0.330	<0.001	−0.212	0.041	−0.118	−0.290–0.047	36

Notes: The covariables included in the analyses were *sex*, *age*, *education*, and *income*. Only significant background variables (*p* < 0.05) are reported. *Age* was significant in the following models with respect to the standardized total effect of X on Y: MA 9:2 (standardized b = −0.198, *p* < 0.001), MA 9:4 (standardized b = −0.140, *p* = 0.016), MA 9:5 (standardized b = 0.134, *p* = 0.030), MA 9:6 (standardized b = 0.206, *p* = 0.001), and MA 9:9 (standardized b = 0.158, *p* = 0.011). *Sex* was significant in the following models with respect to the standardized total effect of X on Y: MA 9:5 (standardized b = −0.133, *p* = 0.027), MA 9:6 (standardized b = −0.127, *p* = 0.046), and MA 9:9 (standardized b = −0.135, *p* = 0.028). *Sex* was also significant in the following models with respect to the standardized direct effect of X on Y: MA 9:5 (standardized b = −0.122, *p* = 0.033), MA 9:6 (standardized b = −0.124, *p* = 0.030), and MA 9:9 (standardized b = −0.121, *p* = 0.034). * Since the percentage of the standardized indirect effect of X on Y relative to the standardized total effect of X on Y is a ratio rather than a proportion (cf. [27]), it can exceed 100%. Table 9 has previously appeared in Andersson [15].

**Table 10:** Mean scores of ratings on the Active Commuting Route Environment Scale (ACRES) for men and women walking to work in the inner urban and suburban areas (mean, SD).

	Outcome Variables		Predictor Variables			
	Hinders–Stimulates Walking	Unsafe–Safe Traffic	Vehicle Speed	Vehicle Flow	Noise	Exhaust Fumes
Inner urban (*n* = 294)	10.4 ± 2.97	10.9 ± 3.40	9.57 ± 3.08	10.2 ± 3.66	9.87 ± 3.28	9.74 ± 3.46
Suburban (*n* = 233)	11.2 ± 3.07	12.2 ± 3.19	7.86 ± 3.79	6.73 ± 4.20	6.64 ± 3.93	6.52 ± 3.92
Ratio inner urban/suburban	0.93	0.89	1.22	1.52	1.49	1.49

**Table 11:** Mean scores of ratings on the Active Commuting Route Environment Scale (ACRES) for men and women cycling to work in the inner urban and suburban areas (mean, SD). Data from [18,19].

	Outcome Variables		Predictor Variables			
	Hinders–Stimulates Cycling	Unsafe–Safe Traffic	Vehicle Speed	Vehicle Flow	Noise	Exhaust Fumes
Inner urban (*n* = 821)	9.16 ± 3.32	8.53 ± 3.69	9.45 ± 2.83	11.1 ± 3.34	9.62 ± 3.04	9.91 ± 3.15
Suburban (*n* = 1098)	11.3 ± 2.84	11.49 ± 2.96	8.40 ± 3.25	7.52 ± 3.95	6.95 ± 3.56	6.72 ± 3.55
Ratio inner urban/suburban	0.81	0.74	1.22	1.48	1.38	1.47

## Data Availability

The data presented in this study are available on request from the corresponding author. The data are not publicly available due to privacy.

## Appendix A

**Table A1:** Descriptive characteristics of the walking commuting trips (mean ± SD, (95% CI) and *n* participants).

	Distance	Duration	Speed	Frequency of Walking Trips in May	Total Number of Walking Trips over the Year *
	**km**	**min**	**km · h^−1^**	**trips · week^−1^**	
Men	3.94 ± 2.50	42.2 ± 22.8	5.42 ± 0.993	4.35 ± 3.57	219 ± 150
	(3.15–4.72)	(34.9–49.6)	(5.09–5.74)	(3.11–5.60)	(163–275)
	41	39	39	34	30
Women	2.92 ± 1.75	32.7 ± 18.6	5.14 ± 0.928	4.63 ± 4.61	227 ± 162
	(2.66–3.17)	(30.0–35.5)	(5.00–5.28)	(3.89–5.37)	(200–255)
	181	175	175	151	135

Notes: The number (*n*) of participants varies across variables and sexes due to missing or incorrectly reported data. * Five females had remarkably high numbers of walking trips per year (range: 736–1183), these outliers were replaced by mean values for the remaining females. After replacing the outliers, the final values were calculated.

**Table A2:** The distribution of time when commuting from home to the place of work or study (*n* participants and percentage within each sex).

	a.m.	5–6	>6–7	>7–8	>8–9	>9–10	>10 a.m.–<5 a.m.
Men (*n* = 43)	*n*	2	7	20	12	1	1
	%	4.7	16.3	46.5	27.9	2.3	2.3
Women (*n* = 187)	*n*	3	34	89	39	9	13
	%	1.6	18.2	47.6	20.9	4.8	7.0

Notes: Four females and two males reported walking in multiple time categories. They were placed in the earliest time category.

**Table A3:** The distribution of time when commuting from the place of work or study to home (*n* participants and percentage within each sex).

	p.m.	2–3	>3–4	>4–5	>5–6	>6–7	>7–8	>8 p.m.– <2 p.m.
Men (*n* = 43)	*n*	5	4	19	3	8	1	3
	%	11.6	9.3	44.2	7.0	18.6	2.3	7.0
Women (*n* = 185)	*n*	3	26	80	56	4	4	12
	%	1.6	14.1	43.2	30.3	2.2	2.2	6.5

Notes: Five females and three males reported that they walked in multiple time categories. They were placed in the earliest time category.

**Table A4:** The VIF, the Cook’s distance and the standardized residuals.

Model	Predictors	Outcomes	VIF		Cook		Std. Residual >(±3 SD)	
			Mean	Maximum	Mean	Maximum	N	Maximum
MRA 5:1 MA 9:1, 9:6, 9:8	Vehicle speed	Noise	1.09	1.19	0.005	0.101	1	3.32
MRA 5:2 MA 9:2, 9:5, 9:7	Vehicle flow	Noise	1.12	1.20	0.005	0.172	3	4.85
MRA 5:3 MA 9:3	Vehicle speed	Exhaust fumes	1.09	1.19	0.005	0.086	1	3.06
MRA 5:4 MA 9:4	Vehicle flow	Exhaust fumes	1.12	1.20	0.005	0.141	5	4.03
MRA 5:5	Noise	Exhaust fumes	1.12	1.20	0.005	0.197	3	−4.18
MRA 5:6 MA 9:2, 9:4	Vehicle speed	Vehicle flow	1.09	1.19	0.004	0.054	–	–
MRA 6:1	Vehicle speed Vehicle flow	Noise	1.38	1.92	0.005	0.189	3	4.90
MRA 6:2	Vehicle speed Vehicle flow	Exhaust fumes	1.38	1.92	0.005	0.130	5	4.01
MRA A5:1	Vehicle speed	Hinders–stimulates walking	1.09	1.19	0.005	0.054	1	−3.06
MRA A5:2	Vehicle flow	Hinders–stimulates walking	1.12	1.20	0.005	0.046	–	–
MRA A5:3 MA 9:5, 9:6, 9:9	Noise	Hinders–stimulates walking	1.12	1.20	0.005	0.044	–	–
MRA A5:4	Exhaust fumes	Hinders–stimulates walking	1.10	1.20	0.005	0.111	–	–
MRA 7:1	Vehicle speed Vehicle flow	Hinders–stimulates walking	1.38	1.92	0.005	0.042	–	–
MRA 7:2	Noise Exhaust fumes	Hinders–stimulates walking	1.76	3.09	0.005	0.095	–	–
MRA 7:3	Vehicle speed Vehicle flow Noise Exhaust fumes	Hinders–stimulates walking	2.34	4.67	0.005	0.087	–	–
MRA A6:1	Vehicle speed	Unsafe–safe traffic	1.09	1.19	0.004	0.077	4	−3.56
MRA A6:2	Vehicle flow	Unsafe–safe traffic	1.12	1.20	0.004	0.060	4	−3.58
MRA A6:3 MA 9:7, 9:8, 9:10	Noise	Unsafe–safe traffic	1.12	1.20	0.004	0.054	2	−3.55
MRA A6:4	Exhaust fumes	Unsafe–safe traffic	1.10	1.20	0.004	0.049	4	−3.61
MRA 8:1	Vehicle speed Vehicle flow	Unsafe–safe traffic	1.38	1.92	0.004	0.067	5	−3.57
MRA 8:2	Noise Exhaust fumes	Unsafe–safe traffic	1.76	3.09	0.004	0.049	3	−3.57
MRA 8:3	Vehicle speed Vehicle flow Noise Exhaust fumes	Unsafe–safe traffic	2.34	4.67	0.004	0.067	5	−3.57
MA 9:1, 9:3	Vehicle flow	Vehicle speed	1.12	1.20	0.005	0.059	–	–
MA 9:9, 9:10	Composite variable	Noise	1.11	1.20	0.005	0.174	1	4.63
Total			1.31	4.67	0.005	0.197	50	4.90

Notes: N = the number of individuals exceeding ± 3 SD. The background variables *sex*, *age*, *education*, and *income* were included in the analyses.

**Table A5:** Relations between the predictor variables and *hinders–stimulates walking* as an outcome.

Model	Outcome	y-Intercept (95% CI)	*p*-Value	Predictor	Unstandardized B (95% CI)	*p*-Value	Adj. R^2^
A5:1	Hinders–stimulates walking	10.1 (7.65–12.5)	<0.001	Vehicle speed	−0.199 (−0.299 to −0.100)	<0.001	0.122
A5:2	Hinders–stimulates walking	11.4 (9.08–13.7)	<0.001	Vehicle flow	−0.292 (−0.380 to −0.205)	<0.001	0.211
A5:3	Hinders–stimulates walking	12.3 (10.1–14.5)	<0.001	Noise	−0.389 (−0.478 to −0.300)	<0.001	0.292
A5:4	Hinders–stimulates walking	11.4 (9.18–13.6)	<0.001	Exhaust fumes	−0.331 (−0.422 to −0.240)	<0.001	0.235

Notes: The background variables *sex*, *age*, *education*, and *income* were included in the analysis. Only significant (*p* < 0.05) background variables are reported. The background variable *sex* was significant in all models: A5:1: unstandardized B = −1.00 (*p* = 0.046); A5:2: unstandardized B = −1.05 (*p* = 0.027); A5:3: unstandardized B = −0.965 (*p* = 0.032); A5:4: unstandardized B = −1.09 (*p* = 0.020). The background variable *age* was significant in model A5:1: unstandardized B = 0.065 (*p* = 0.001); A5:2: unstandardized B = 0.042 (*p* = 0.030); A5:4: unstandardized B = 0.048 (*p* = 0.010).

**Table A6:** Relations between the predictor variables and *unsafe–safe traffic* as an outcome.

Model	Outcome	y-Intercept (95% CI)	*p*-Value	Predictor	Unstandardized B (95% CI)	*p*-Value	Adj. R^2^
A6:1	Unsafe–safe traffic	14.6 (12.1–17.2)	<0.001	Vehicle speed	−0.254 (−0.360 to −0.147)	<0.001	0.076
A6:2	Unsafe–safe traffic	15.0 (12.4–17.5)	<0.001	Vehicle flow	−0.262 (−0.359 to −0.164)	<0.001	0.097
A6:3	Unsafe–safe traffic	14.8 (12.3–17.4)	<0.001	Noise	−0.261 (−0.366 to −0.155)	<0.001	0.082
A6:4	Unsafe–safe traffic	14.4 (11.8–16.9)	<0.001	Exhaust fumes	−0.235 (−0.340 to −0.131)	<0.001	0.067

Notes: The background variables *sex*, *age*, *education*, and *income* were included in the analysis. Only significant (*p* < 0.05) background variables are reported. There were no significant background variables.
